# High pesticide risk to honey bees despite low focal crop pollen collection during pollination of a mass blooming crop

**DOI:** 10.1038/srep46554

**Published:** 2017-04-19

**Authors:** Scott H. McArt, Ashley A. Fersch, Nelson J. Milano, Lauren L. Truitt, Katalin Böröczky

**Affiliations:** 1 Department of Entomology, Cornell University, Ithaca, NY 14853, USA; 2 Department of Ecology and Evolutionary Biology, Cornell University, Ithaca, NY 14853, USA

## Abstract

Honey bees provide critical pollination services for many agricultural crops. While the contribution of pesticides to current hive loss rates is debated, remarkably little is known regarding the magnitude of risk to bees and mechanisms of exposure during pollination. Here, we show that pesticide risk in recently accumulated beebread was above regulatory agency levels of concern for acute or chronic exposure at 5 and 22 of the 30 apple orchards, respectively, where we placed 120 experimental hives. Landscape context strongly predicted focal crop pollen foraging and total pesticide residues, which were dominated by fungicides. Yet focal crop pollen foraging was a poor predictor of pesticide risk, which was driven primarily by insecticides. Instead, risk was positively related to diversity of non-focal crop pollen sources. Furthermore, over 60% of pesticide risk was attributed to pesticides that were not sprayed during the apple bloom period. These results suggest the majority of pesticide risk to honey bees providing pollination services came from residues in non-focal crop pollen, likely contaminated wildflowers or other sources. We suggest a greater understanding of the specific mechanisms of non-focal crop pesticide exposure is essential for minimizing risk to bees and improving the sustainability of grower pest management programs.

Crop pollination by insects is worth over $15 billion/year to the US economy^1^ and approximately $170 billion/year globally^2^. A frequently cited figure is that one third of our food is dependent on pollinating insects^3^. Yet managed and wild pollinators are currently experiencing alarming loss rates and declines worldwide. For example, in 2014–2015 the average loss rate for honey bee hives in the US was 42.1%^4^. Similarly, many species of wild bees have experienced well documented range contractions and extinctions in North America, Europe and elsewhere over the past few decades^5^,^6^,^7^,^8^. These losses of managed and wild bees are coming at a time when global agricultural dependence on pollinators continues to increase each year^9^.

Pollinators are at risk from several factors, including parasites and pathogens, lack of floral resources, environmental toxins and other stresses^10^. Among these factors, there is considerable interest regarding the effect that pesticides are having on bees. A growing number of studies confirm that pesticide residues are commonly found in pollen and wax in honey bee colonies near agricultural settings^11^,^12^,^13^,^14^,^15^,^16^. Residues are often found at levels known to influence susceptibility to parasites and pathogens^17^,^18^,^19^, foraging behaviors^20^,^21^, and growth and survival of bees^20^,^22^,^23^,^24^, suggesting a potential for detrimental effects.

It is generally assumed that bees are exposed to pesticides during crop pollination, yet surprisingly little is known regarding how focal crop pollen collection is related to pesticide exposure, how landscape context influences crop pollen collection, and whether the magnitude of pesticide risk to bees is at levels warranting concern. Pettis, *et al*.^12^ found that focal crop pollen collection by honey bees was generally high in almond and apple (both > 70%), but below 10% in blueberry, cranberry, cucumber, watermelon and pumpkin plantings. Similarly, Odoux, *et al*.^25^ and Garbuzov, *et al*.^26^ found that honey bee foraging on neonicotinoid-treated oilseed rape was never more than 28% and 17%, respectively, and the magnitude of rape pollen collection was likely driven in part by landscape context. Such observations have prompted speculation that pesticide exposure may be minimized when honey bees providing pollination services forage primarily on non-focal crop sources of pollen and nectar^15^, whose quality and diversity may influence the effects of pesticides^27^,^28^. However, pesticide residues in bee-collected pollen can be high despite non-crop sources accounting for the majority of pollen^16^. These results suggest either that non-crop sources of pollen may be generally contaminated with pesticide residues in agricultural landscapes^29^,^30^,^31^,^32^, or small amounts of focal crop pollen collection by bees can lead to substantial pesticide exposure.

To understand mechanisms of how bees are exposed to pesticides during crop pollination, and when pesticide exposure represents a substantial risk, comparative studies that simultaneously assess grower spray practices, bee foraging decisions, and in-hive pesticide residue levels across a gradient of landscape complexity must be conducted. Yet such studies do not exist to our knowledge. In this study, we use a network of 120 experimental honey bee colonies placed in 30 apple orchards to address three related questions: (1) How does landscape context govern focal crop foraging by honey bees during pollination of apple, a mass-blooming crop, (2) Is focal crop pollen foraging related to pesticide residues in recently accumulated beebread and/or pesticide risk, as measured by the Pesticide Use Index (PUI)^33^ and Pollen Hazard Quotient (PHQ)^34^, and (3) Given different on-farm pest management practices and landscape context surrounding farms, when do these factors lead to substantial pesticide risk to bees?

## Methods

### Field experimental design

In April 2015, we purchased 120 5-frame nucleus colonies of honey bees from a local commercial beekeeper. All nucleus colonies were transferred into new 10-frame equipment with plastic foundation and allowed to draw comb for two weeks in a common location (Dyce Lab for Honey Bee Studies, Ithaca, NY: 42.466118, –76.446211). During this time, all colonies were assessed for strength and queen status. Of the 120 colonies, 5 were determined to be queenless and replaced with new queens from the same genetic source. Following assessments, frames were redistributed among colonies such that all colonies had a similar composition of brood, bees, pollen and nectar prior to enrollment in the field experiment.

Corresponding with the first sign of bloom in each orchard (between May 7–11, 2015), we transferred 4 colonies into each of 30 apple orchard sites in western and central New York (Table S1). Colonies remained at orchard sites for the duration of bloom, mimicking how beekeepers typically provide pollination services for pollination-dependent crops such as apple. At the end of the bloom period at each site (May 16–22), we collected ~3 g recently accumulated beebread from each colony, ensuring to collect beebread from frames of newly drawn comb so any possible pesticide residues from old comb were avoided. Three grams beebread represents ~70–100 pollen cells, often the entirety or majority of freshly collected beebread on a frame, and is the typical mass of pollen used for pesticide residue analyses in similar ecotoxicology studies^11^,^12^,^35^. Since beebread is consumed ~72–96 hours after it is collected on average, with ~20% consumption occurring beyond 96 hrs^36^, using freshly collected beebread integrates several days of pollen collected during the 10-day pollination period at each site. All beebread was placed on dry ice immediately and stored in a −80 C freezer until laboratory analyses.

### Pollen identification

Pollen was identified to the lowest taxonomic level possible using standard microscopy techniques. Briefly, each 3 g sample of beebread was homogenized for 5 minutes in a shallow dish using a gloved hand. This homogenization process was gentle enough to ensure individual pollen grains were not damaged, yet thorough enough to ensure mixing of the different types of pollen. Mixing was easily observed as the different colors of pollen became a uniform mass following only a minute or two of homogenization, after which we continued to homogenize the sample by hand for several minutes. We created a 24 mg pooled subsample for each site by taking 6.0 ± 0.3 mg of each homogenized sample corresponding to each colony (*n* = 4 colonies per site). We added 500 μl DI water, vortexed for 15 secs, sonicated for 2 min, then centrifuged for 3 min at 16,000 g. Water was removed, 200 μl 95% ethanol was added, then the sample was vortexed and sonicated as previously. Ten μl of the resulting suspension was added to a clean glass slide along with 40 μl Calberla’s solution, mixed, allowed to sit at 25 C for 20 mins, then covered with a coverslip and sealed with clear nail polish after 24 hrs.

**Figure 1:**
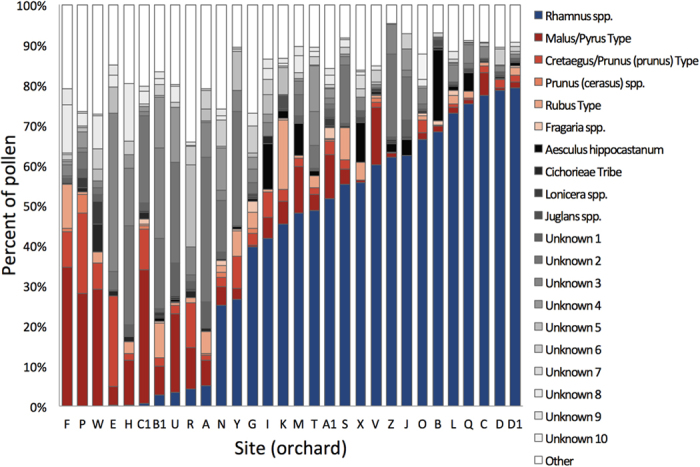
Pollen collected by honey bees across 30 apple orchard sites during bloom. Ten pollen types were identified using reference samples from field collections, an online pollen reference library, http://blogs.cornell.edu/pollengrains/, and relevant literature^38^. Ten additional pollen morphotypes (Unknown 1–10) were found at greater than 3% relative abundance at a minimum of one site and therefore quantified; any grain not fitting one of the types and therefore below 3% relative abundance was determined to be sporadic and classified as Other^37^. Percentages of each pollen type are shown in Table S5.

One slide was analyzed per site using a transect approach and 400× magnification on a compound microscope. Briefly, transects were initiated in a random location on the margin of each slide and all pollen grains that were entirely in the field of view were counted and classified until 300 pollen grains total was reached. Pollen grains were classified according to 20 morphotypes that were found to be greater than 3% relative abundance on any of the individual slides (see Fig. 1). Any grain not fitting one of the types and therefore below 3% relative abundance was determined to be sporadic^37^ and put into the “other” category. Representative samples for each morphotype were compared to reference samples collected during the study, an open access pollen reference library made for plants blooming around our orchard sites (http://blogs.cornell.edu/pollengrains/) and relevant literature^38^.

### Landscape characterization

**Figure 2:**
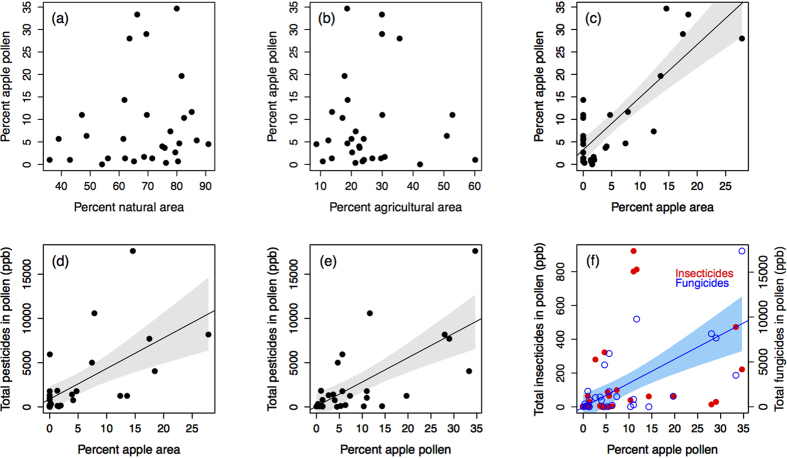
Landscape characteristics, percent focal crop (apple) pollen collection by honey bees and pesticide residues across the 30 orchard sites. Percent natural area and agricultural area (**a**,**b**) did not predict apple pollen collection, while percent apple area in the landscape (**c**) did predict apple pollen collection. Percent apple area (**d**) and apple pollen (**e**) predicted total pesticide residues in pollen collected by honey bees. This relationship was driven primarily by fungicides (**f**, open blue points); there was no relationship between percent apple pollen and total insecticides (**f**, solid red points). 95% confidence intervals shown for all significant correlations.

We used a geographical information system, ArcGIS v10 (Environmental Systems Research Institute, Redlands, CA, USA), and the Cropland Data Layer^39^ (30 m resolution), provided by USDA NASS, to quantify percent natural area, agricultural area and focal crop (apple) area at each hive location using three spatial buffers (1000, 2000 and 3000 m). These buffers were chosen because they span typical median foraging radii of honey bees, including the region of our study in upstate New York^40^,^41^. We considered natural areas to be comprised of all forest types, shrubland, grassland/pasture, developed open, developed low, all wetland types, fallow/idle land and clover/wildflowers. We classified agricultural area as all crop types, including corn, soybeans, barley, wheat, rye, oats, speltz, alfalfa, hay, buckwheat, beans, tomatoes, hops, cherries, apples, grapes, Christmas trees, grass, triticale, plums, squash, pumpkins, cabbage, cauliflower, millet, onion, cucumber, peas, carrots, strawberries, turnips, lettuce, potatoes, sunflower and sorghum. We classified apple area as the percent of apple compared to all other land use types. We determined that 3000 m was the scale at which percentage of natural area, agricultural area, and apple area provided the best model fit to the data (based on AIC values, see Table S2). Thus, we conducted all landscape analyses using the 3000 m buffer (Fig. 2).

### Pesticide analyses

At each orchard, we assessed pesticide risk to bees using two methods. First, we collected spray record information during apple bloom (May 7–22, 2015) in order to calculate Pesticide Use Index (PUI)^33^, which was modified slightly from the original reference (see below). Pesticide Use Index was calculated using the equation:


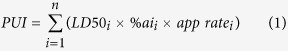


where, *LD50* is the toxicity to honey bees according to each pesticide’s contact LD_50_ value, %*ai* is the percent of active ingredient, and *app rate* is the application rate in quantity per hectare. We used LD_50_ information in place of the original 1–5 toxicity scale proposed for measuring pesticide toxicity^42^ to more accurately represent the broad toxicity range of pesticides sprayed in orchards (i.e. contact LD_50_ values ranging continuously over 5 orders of magnitude, from 0.003 to 312 ug/bee; Table S3). We calculated PUI using all apple bloom spray record data (Table S3, Fig. S1b,c), and using only the compounds quantified in pollen (see below, Fig. S1d,e).

Next, we quantified 25 pesticides (14 insecticides, 10 fungicides, 1 herbicide; see Table 1) from beebread using a modified QuEChERS extraction protocol^43^ and LC-MS/MS system for quantification. This analysis captured 16 of the 30 pesticides sprayed during bloom across the 30 sites, including 9 of 12 insecticides, 7 of 16 fungicides, and 0 of 2 herbicides (Table S3). Compounds sprayed during bloom but omitted from the residue analysis were either impossible to quantify using LC-MS/MS (e.g. captan, mancozeb), unable to be quantified via our extraction/quantification protocol (e.g. glyphosate, potassium bicarbonate), or exhibited unreliable quantification parameters and were therefore removed from the multi-residue analysis (e.g. pyrethrins). We also quantified 9 common pesticides that were not sprayed during bloom at the 30 sites, including 5 insecticides (chlorantraniliprole, clothianidin, cyfluthrin, fenpyroximate, imidacloprid), 3 fungicides (fluxapyroxad, myclobutanil, boscalid) and 1 herbicide (atrazine).

Extraction and purification of pesticides from beebread samples occurred by first weighing 3 ± 0.004 g of mixed beebread samples from each site (4 × 0.75 ± 0.001 g samples from each colony were combined). We hydrated samples with 15 mL DI H_2_O for 30 mins, then added the extraction solvent mix (15 ml acetonitrile, 150 μl acetic acid, 6 g MgSO_4_, 1.5 g sodium acetate) and homogenized samples at 5 m/s for 1 min via a bead ruptor (Omni International, Kennesaw, GA). We then centrifuged samples (4000 g, 10 min) and conducted two sequential solid phase extraction (SPE) clean-up steps. First, we added 12 ml supernatant to a mix of 1.5 g MgSO_4_, 0.5 g PSA, 0.5 C-18 g silica, vortexed, then placed on a reciprocal shaker for 1 hr at 300 revs/min. We then centrifuged samples (4000 g, 10 min) and conducted a second SPE clean-up by adding 8 ml supernatant to 4 ml acetonitrile, 1.5 g MgSO_4_, 0.5 g PSA, 0.5 g C-18 silica, vortexed, then placed on a reciprocal shaker for 1 hr at 300 revs/min. Following centrifugation (4000 g, 10 min), we added 7 mL supernatant to a glass tube and concentrated the extract to <1 ml using a Nitrogen evaporator (Organomation, Berlin, MA). Final solutions were brought up to 1 ml with acetonitrile, filtered via a 0.21 μm Costar spin-filter (Corning Inc., Corning, NY) and 100 μl was added to a 2 ml screw-cap vial for analysis.

Samples were analyzed in a triple quadrupole LC-MS/MS system (an Accela liquid chromatograph coupled with a TSQ Quantum Access mass spectrometer; Thermo Scientific) equipped with a C18 reversed-phase column (Kinetex 2.6 μm EVO C18, 150 × 2.10 mm; Phenomenex). The mobile phase solutions consisted of 5 mM ammonium formate and 0.05% formic acid in either acetonitrile (LC-MS grade; Fisher Scientific) with 10% MilliQ water (A) or in MilliQ water (B). A gradient of 5% A for 2 min, to 25% A in 2 min, and finally to 100% A in 25 min (maintained for 3 min) was run at a flow rate of 200 μl/min. Compounds were ionized in the electrospray interface in the positive mode (ESI+) and fragmented further to allow detection via selected ion monitoring (SRM). Detection parameters were optimized for each analyte by injecting a 5 or 50 ng/μl solution in acetonitrile with 0.1% formic acid into the mobile phase flow using the auto-loop injection mode of the instrument. Spray voltage, sheath gas pressure, and ion sweep pressure were the same for all the analytes (4500 V, 40 (arbitrary), and 4 (arbitrary), respectively) while individual tube lens offset and collision energy values were used for the transitions. The collision gas was argon at a pressure of 1.5 mTorr. For each analyte, the two most abundant transitions were monitored and only the most abundant one was used for quantitation. We included two spiked samples (at 3 ppb and 30 ppb of all compounds analyzed) and one blank sample using pesticide-free honey bee pollen (CC Pollen Co., Phoenix, AZ). Due to the large variation in the chemical properties of the analytes, external calibration was used. Seven calibration solutions were prepared in extracted pesticide-free honey bee pollen in the range of 0.0002−5 ng/μl. Analyte concentrations in the final extracts were determined based on the fitted curves and back-calculated for a ppb in sample value taking extraction losses into consideration. Limit of quantitation was determined based on the lowest working calibration point.

To estimate pesticide risk from residues in the beebread, we computed a Pollen Hazard Quotient (PHQ)^34^. We first computed a raw PHQ by summing each pesticide residue (ng/g pollen) divided by the respective honey bee LD_50_ (ug/bee):


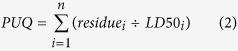


**Figure 3:**
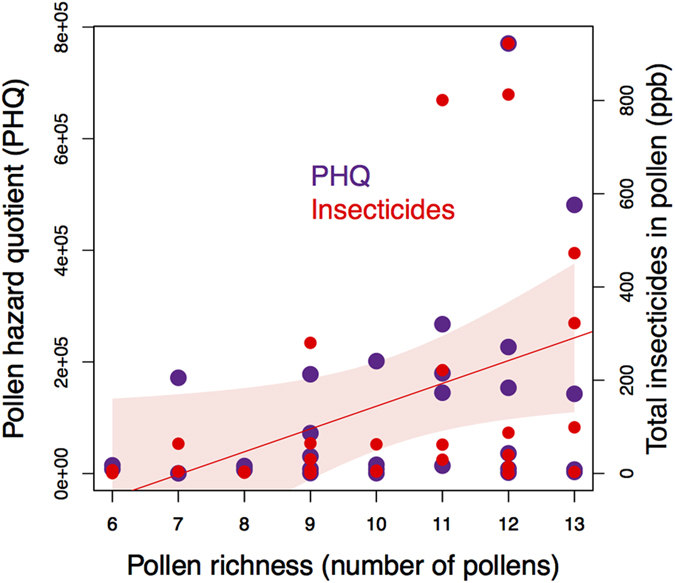
Relationship between pollen richness (the number of pollens in recently accumulated beebread) and total insecticide residues (red) and Pollen Hazard Quotient (PHQ; purple)^34^ across the 30 orchard sites. 95% confidence interval shown for significant correlation.

Toxicity data for honey bees were obtained from the Tomlin Pesticide Manual^44^, the ECOTOX database of the United States Environmental Protection Agency (http://cfpub.epa.gov/ecotox/) and the AgriTox Database of the French government (http://www.agritox.anses.fr/index.php). The raw PHQ was used in two ways: 1) to assess risk from individual pesticides across all sites (Table 1), and 2) to estimate risk at each of the 30 sites from all pesticides found in beebread at that site (Fig. 3, Fig. S1).

**Figure 4:**
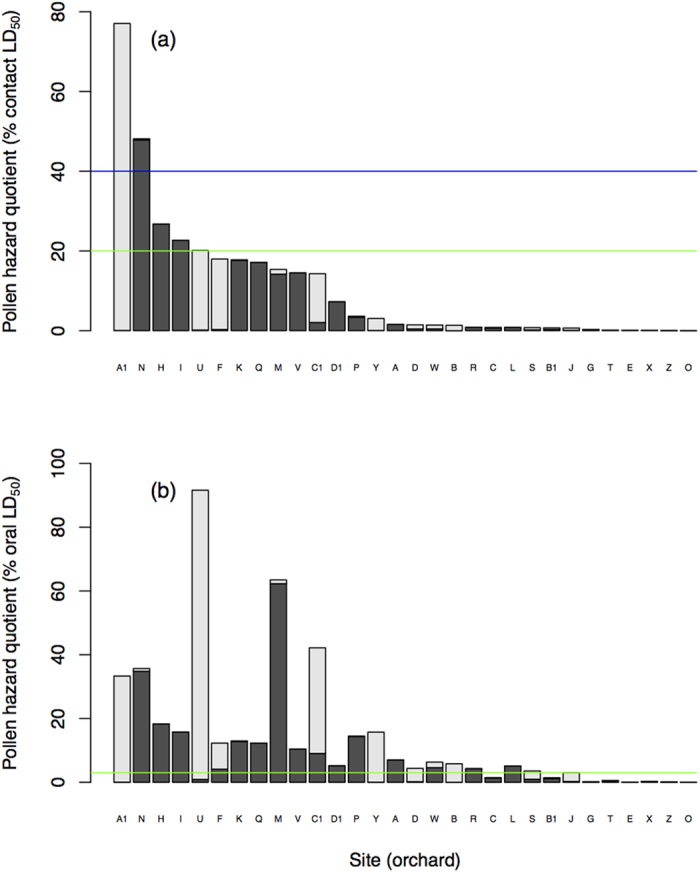
Contact (**a**) and oral (**b**) pollen hazard quotients (expressed as percent of total LD_50_) in recently accumulated beebread collected from hives at the 30 orchard sites. Light gray portion of bars represent proportion of hazard quotient attributed to pesticides that were sprayed during bloom at each site. Dark gray portion of bars represent proportion of hazard quotient attributed to pesticides that were not sprayed during bloom at each site. Solid blue line in (**a**) corresponds to the current United States Environmental Protection Agency level of concern for acute contact exposure (Tier 1 risk quotient = 0.4)^45^. Solid green line in (**a**) corresponds to the European Food Safety Authority (EFSA) level of concern for acute contact exposure (exposure/toxicity = 0.2)^46^. Solid green line in (**b**) corresponds to EFSA level of concern for chronic oral exposure (exposure/toxicity = 0.03)^46^. See *Materials and Methods* for details regarding pollen hazard quotient calculations.

Next, to relate PHQ values to United States Environmental Protection Agency (EPA)^45^ and European Food Safety Authority (EFSA)^46^ levels of concern for pesticide risk, we related PHQ to percent of honey bee LD_50_ values (Fig. 4). To do so, we assumed 1) all pesticides interact additively, 2) an average adult bee weight of 100 mg, 3) acute contact exposure occurs from contacting a body-weight equivalent of pollen over 2–4 days^15^ (i.e., the typical duration of a laboratory contact LD_50_ study), and 4) chronic oral exposure occurs from ingesting 9.4 mg pollen/day^47^ for the median duration of apple bloom and pollination in the 30 orchards (10 days), corresponding to an LC_50_ 10-day chronic exposure^46^. Contacting 100 mg pollen over 2–4 days is likely an underestimate for pollen-foraging adult worker bees, who typically return from individual foraging bouts carrying 20 mg pollen on average^48^. This is also likely an underestimate for pollen-provisioning nurse bees, who feed 2.7 mg pollen/day to each individual larva on average^47^. Pollen contact estimates for other castes are more difficult to determine due to lack of data. However, given the constant movement of pollen in the hive, not to mention contact with honey, wax and other potentially contaminated bee products, we suggest the contact estimate of Frazier *et al*.^15^ is reasonable.

Because the Tier 1 EPA risk quotient = 0.4 for acute contact exposure^45^, we present this reference value in relation to our contact PHQ results (blue line in Fig. 4a). Because the EFSA ratio of exposure and toxicity, ETR = exposure/toxicity = 0.2 for acute contact exposure^46^, we also present this reference value in relation to our contact PHQ results (green line in Fig. 4a). In addition, because EFSA considers LC_50_ 10-days to represent chronic oral exposure risk^46^, which corresponds to the 10-day pollination period in our study, we present the ETR trigger value of 0.03 (green line in Fig. 4b). EPA risk assessment for chronic exposure relies on no observable adverse effect concentration (NOAEC) data, which exist for only a fraction of the compounds quantified in our study, limiting the utility of EPA’s chronic risk metric for our data.

We used linear models and general linear models using the lm and glm functions in R (version 3.2.3)^49^ to test for relationships between landscape variables, pollen abundance and composition, and pesticide metrics (pesticide residues, PUI and PHQ).

## Results

Across the 30 apple sites, we found 20 major types of pollen collected by honey bees during apple bloom (Fig. 1, Table S5). The dominant pollen type was *Rhamnus* spp. (buckthorn), which was collected at 27 of 30 sites and comprised 38.6% of pollen on average (range: 0–79.3%). While focal crop *Malus* (apple) pollen was collected at 29 of 30 sites, it comprised only 8.7% of pollen on average (range: 0–34.7%). *Cretaegus/Prunus* (hawthorn/plum) comprised 4.3% of pollen on average and was present at 27 sites, while *Rubus* spp. (blackberry/raspberry) comprised 3.0% of pollen on average and was present at 26 sites. Two unknown pollen types (Unknown 2 and Unknown 3) comprised over 5% of pollen. Unknown 2 comprised 8.1% on average (24 sites) and Unknown 3 comprised 5.3% (27 sites). Other pollens were more variable among sites, including Unknown 4 comprising 2.4% on average (20 sites)*, Aesculus hippocastanum* (horse chestnut) comprising 2.2% on average (18 sites), *Chicorieae* (dandelion tribe) comprising 0.7% on average (10 sites), *Lonicera* spp. (honeysuckle) comprising 0.6% on average (18 sites), *Fragaria* spp. (strawberry) comprising 0.4% on average (13 sites), and *Prunus cerasus* spp. (cherry) comprising 0.4% on average (10 sites).

Because foraging choices by honey bees are dictated in part by landscape context^50^, we tested whether the amount of natural area, agricultural area or focal crop (apple) area predicted focal crop pollen collection by bees at each site. Percent natural area and agricultural area did not influence focal crop pollen collection (Fig. 2a and b, *P* > 0.4), while percent of apple grown in the landscape was a strong predictor of percent apple pollen collection (Fig. 2c; *F*_1,28_ = 65.1, *P* < 0.001, *R*^*2*^ = 0.67).

Next, because pesticide residues are often found in the pollen collected by bees near mass blooming crops^11^,^12^,^13^,^14^,^15^,^16^, we tested whether percent focal crop pollen and apple crop area in the landscape predicted pesticide concentrations in beebread. Using our 25-compound analysis, we found that percent apple area in the landscape predicted total pesticides in beebread (Fig. 2d; *F*_1,27_ = 17.2, *P* < 0.001, *R*^*2*^ = 0.37) and percent apple pollen collected was a stronger predictor of total pesticides in beebread (Fig. 2e; *F*_1,27_ = 25.7, *P* < 0.001, *R*^*2*^ = 0.47). When broken down by type of pesticide, we found a significant relationship between percent apple pollen and total fungicides (Fig. 2f, blue; *F*_1,27_ = 24.9, *P* < 0.001, *R*^*2*^ = 0.46) but no relationship between percent apple pollen and total insecticides (Fig. 2f, red; *F*_1,27_ = 2.0, *P* = 0.17, *R*^*2*^ = 0.03). The fungicide results are consistent with grower pest management practices during bloom at the 30 orchards in the study, which were predominantly fungicide sprays (Table S3).

We found that fungicides accounted for 94% of total residues (in ppb) in beebread (Table 1). However, when we calculated pesticide risk via contact and oral pollen hazard quotients (PHQ), we found that insecticides represented the majority of pesticide risk to bees, accounting for 98.4% of contact and 97.7% of oral PHQ (Table 1). Thus, we investigated further the drivers and magnitude of pesticide risk, particularly due to insecticides, across our 30 orchard sites. Insecticide residues were consistent predictors of pesticide risk to bees across sites via PHQ (Fig. S1a red: *F*_1,27_ = 28.6, *P* < 0.001, *R*^*2*^ = 0.50), while there was no relationship between fungicides and PHQ across sites (Fig. S1a blue: *F*_1,27_ = 0.3, *P* = 0.59, *R*^*2*^ = 0.01). There was no relationship between the spray record-based Pesticide Use Index (PUI) and insecticide (Fig. S1b red; *F*_1,27_ = 1.5, *P* = 0.14, *R*^*2*^ = 0.05) or fungicide (Fig. S1b blue; *F*_1,27_ = 0.9, *P* = 0.34, *R*^*2*^ = 0.03) residues in beebread when all compounds sprayed during bloom were used to calculate PUI. When only the 25 compounds quantified in beebread were used to calculate PUI, we found a positive relationship between PUI and insecticide residues (Fig. S1d red; *F*_1,27_ = 2.7, *P* = 0.013, *R*^*2*^ = 0.18) and no relationship between PUI and fungicide residues (Fig. S1d blue; *F*_1,27_ = 1.5, *P* = 0.14, *R*^*2*^ = 0.05). Similarly, we found no relationship between the PUI and PHQ risk metrics across our 30 sites when all compounds sprayed during bloom were used to calculate PUI (Fig. S1c; *F*_1,27_ = 1.8, *P* = 0.09, *R*^*2*^ = 0.07), and a positive relationship between PUI and PHQ when only the 25 compounds quantified in beebread were used to calculate PUI (Fig. S1e; *F*_1,27_ = 2.4, *P* = 0.021, *R*^*2*^ = 0.15).

We found no relationship between percent focal crop (apple) pollen collected by bees and either PHQ (*F*_1,27_ = 0.8, *P* = 0.37, *R*^*2*^ = 0.02), PUI (*F*_1,27_ = 0.5, *P* = 0.51, *R*^*2*^ = 0.01) or total insecticides (Fig. 2f, red; *F*_1,27_ = 2.0, *P* = 0.17, *R*^*2*^ = 0.03). Furthermore, we found no relationship between percent focal crop (apple) in the landscape and PHQ (*F*_1,27_ = 0.2, *P* = 0.67, *R*^*2*^ = 0.01), PUI (*F*_1,27_ = 0.0, *P* = 0.98, *R*^*2*^ = 0.00) or total insecticides (*F*_1,27_ = 0.5, *P* = 0.51, *R*^*2*^ = 0.01). Instead, we found that pollen richness (i.e., the number of pollen types) predicted total insecticides in beebread (Fig. 3 red; *P* = 0.036, *R*^*2*^ = 0.12) and marginally predicted PHQ (Fig. 3 purple; *P* = 0.072, *R*^*2*^ = 0.09). Pesticides that were not sprayed during bloom were found in beebread at 28 of 30 sites, averaging 2.8 novel pesticides per site (Table S4). Overall, 64% of the pesticides we detected in beebread were not sprayed at the respective sites during apple bloom. Because several of these pesticides were highly toxic insecticides, pesticides that were not sprayed during apple bloom accounted for 62% and 66% of contact and oral pesticide risk, respectively, across the 30 sites (range: 0–100%, Table S4, Fig. 4).

Finally, we investigated whether the levels of pesticides found in beebread represented a substantial risk to bees. We found that risk from acute contact exposure was above the United States Environmental Protection Agency level of concern at 2 of 30 orchard sites (Tier 1 risk quotient = 0.4)^45^, and above the European Food Safety Authority (EFSA) level of concern at 5 of 30 orchard sites (exposure/toxicity = 0.2)^46^ (Fig. 4a). We found that risk from chronic oral exposure was above the EFSA level of concern at 22 of 30 orchards (exposure/toxicity = 0.03)^46^ (Fig. 4b). Thus, bees at the majority of the orchard sites experienced pesticide risk exceeding a regulatory agency-determined level of concern for acute or chronic exposure during the apple bloom period.

## Discussion

To our knowledge, our results provide the first evidence that pesticide exposure to bees during pollination of a mass blooming crop is linked to both focal crop pollen collection and crop density in the landscape. Thus, landscape context is an important factor influencing pesticide exposure to bees pollinating a mass blooming crop. However, we also found that pesticide risk to bees (as measured by Pesticide Use Index and Pollen Hazard Quotient) was decoupled from focal crop pollen collection and landscape context. Our results suggest the majority of pesticide risk came from non-focal crop pollen sources and pesticides that were not sprayed during the apple bloom period. Because we found that risk from acute or chronic pesticide exposure was above regulatory agency-determined levels of concern at the majority of the orchard sites in our study, this latter result may have important implications for grower pest management practices that seek to minimize risk to bees.

Insecticides in pollen and nectar of non-crop plants at field margins have been found at levels that pose substantial risk to bees^29^,^30^,^31^,^32^. These studies investigating field margins have focused primarily on neonicotinoid insecticides, which are persistent in soil and accumulate in pollen and nectar via their systemic activity in plants^51^. Such a mechanism of exposure may apply to the pesticide with the greatest total oral exposure risk to bees in our study, the neonicotinoid thiamethoxam (Table 1). Thiamethoxam was sprayed at one orchard during bloom, yet was found in recently accumulated beebread at 5 of 30 orchard sites, none of which were the orchard where it was sprayed during bloom (Table 1, Tables S3, S4). Thiamethoxam was not sprayed immediately prior to bloom at any of our sites, though it was used at several orchards in years prior to our study. Similarly, the pesticide with the greatest total contact exposure risk to bees in our study, cyfluthrin, was not sprayed at any orchards during bloom, yet was found in recently accumulated beebread at 6 of 30 orchard sites (Table 1, Table S3). Cyfluthrin was not sprayed immediately prior to bloom at any sites, though it was used at several orchards in years prior and at other times during the growing season (hence why it was included in our 25-compound laboratory analysis). Cyfluthrin is not a systemic insecticide and is sensitive to breakdown by sunlight, yet its persistence on plant material has been observed at up to 52 weeks^52^.

Further supporting the role of non-focal crop avenues of pesticide risk to bees during the apple pollination period, three of our orchards (I, V and X) did not spray any pesticides during bloom, yet pesticide residues were found in recently accumulated beebread at all three orchards (Table S4). Bees at sites I and X collected only 0.3 and 0.7% apple pollen, respectively, yet had detectable levels of 1 and 5 pesticides. Bees at site V collected a greater proportion of apple pollen (14.3%) and 3 pesticides were detected, yet no pesticides were sprayed at this site during bloom or for at least the 3 years preceding our study (McArt, personal communication). Recently accumulated beebread at sites I and V was found to have pesticide risk above the European Food Safety Authority level of concern for either acute contact (I) and chronic oral (I and V) exposure (Fig. 4). Taken together, these results are in line with a recent study that found high levels of pesticides in honey bee-collected pollen in agricultural and semi-natural landscapes, even though the majority of pollen was collected from non-crop flowers 16.

Some risk from pesticides sprayed during bloom, but not quantified in beebread via our 25-compound analysis, could have been missed. However, this is unlikely to alter conclusions regarding non-focal crop sources of risk for at least three reasons. First, of the 14 compounds sprayed during bloom but not quantified in beebread, 11 are fungicides or herbicides. All fungicides and herbicides sprayed during bloom have high LD_50_ values for honey bees (Table S3) and are therefore unlikely to contribute substantially to risk. For example, fungicides accounted for 94% of total residues in beebread, but only 1.6–2.3% of risk as measured by contact or oral PHQ. Second, while we were unfortunately unable to quantify in beebread 3 insecticides sprayed during bloom (emamectin benzoate, lambda-cyhalothrin and pyrethrins), these compounds were sprayed at only 4 sites. We quantified the other 9 insecticides that were sprayed during bloom (acetamiprid, carbaryl, indoxacarb, phosmet, spinetoram, spinosad, thiacloprid and thiamethoxam), which were sprayed at a total of 18 sites. Thus, while we could potentially have missed some positive detections of insecticides sprayed during bloom, our data likely captures the great majority of insecticide residues that originate from during-bloom sprays. Third, we quantified in the beebread 5 insecticides (chlorantraniliprole, clothianidin, cyfluthrin, fenpyroximate and imidacloprid), 3 fungicides (boscalid, fluxapyroxad, myclobutanil) and 1 herbicide (atrazine) that were not sprayed at any sites during bloom, finding positive detections of 2 insecticides (cyfluthrin and fenpyroximate) at 7 sites, 2 fungicides (fluxapyroxad and myclobutanil) at 13 sites, and 1 herbicide (atrazine) at 6 sites. Cyfluthrin alone accounted for 48% and 25% of total contact and oral pesticide risk in the study, respectively (Table 1). Considering that >150 additional insecticide, fungicide and herbicide compounds have been found in beebread^14^,^35^, but weren’t screened for in our study, our data may in fact overestimate the proportion of pesticide risk that comes from during-bloom sprays.

Though our results suggest the greatest pesticide risk to bees came from non-focal crop pollen sources, our data cannot determine the exact mechanism or mechanisms of exposure. Because several of the identified pollen types were likely from plants observed surrounding orchards (e.g., *Rhamnus* spp., *Rubus* spp., *Prunus* spp.) or lanes between orchard rows (*Chicorieae, Fragaria* spp.), it is possible that pesticide drift from sprays or leaching into the soil resulted in residue accumulation in non-focal crop flowers. However, because residues were found in beebread at orchard sites that did not spray pesticides, this mechanism likely does not account for all residues leading to high pesticide risk. Furthermore, we note that the spray-based PUI risk metric explained only 7% and 15% of variation in the pollen-based PHQ risk metric for all pesticides sprayed and the specific compounds we quantified from pollen in the laboratory, respectively (Fig. S1d,f). Thus, between 85–93% of pesticide risk was not accounted for by pesticide sprays on the focal crop or drift that may have occurred into field margins during bloom. Other sources of residues were therefore likely, yet the origin of those residues cannot be determined from our data.

One of the underlying assumptions of pesticide risk metrics such as PHQ and PUI is that pesticides interact additively (i.e., synergism or antagonism between pesticides does not lead to greater or less toxicity). In fact, the literature on this topic to date suggests synergism between pesticides is common, often resulting in increased toxicity compared to additive predictions. For example, while fungicides are relatively non-toxic to honey bees on their own, studies have found that ergosterol biosynthesis inhibiting (EBI) fungicides can increase the toxicity of pyrethroid insecticides up to 1000-fold^53^,^54^. Interactions are also known to occur between EBI fungicides and neonicotinoids, including up to an 8-fold increase in toxicity of thiamethoxam^55^. While ~98% of the pesticide risk in our study (via contact and oral PHQ) was due to insecticides (Table 1), 94% of pesticide residues (in ppb) were fungicides, which is similar to previous studies that have quantified pesticide residues in honey bee-collected pollen in agricultural settings^11^,^12^,^35^. Of the fungicides quantified in our study, we note that difenoconazole and fenbuconazole, both EBI fungicides, were detected at 22 of our 30 orchard sites, and at 14 of these orchards we also detected pyrethroids or neonicotinoids (Table 1). Thus, our data suggest that, if anything, we are underestimating the pesticide risk to bees in this study.

Because our results show that pesticide risk to honey bees during crop pollination can be substantial, we suggest that further effort be placed in understanding where bees come into contact with high-risk pesticides in agricultural settings. Our results suggest the majority of risk came from pesticides that were not sprayed during bloom and non-focal crop sources. Whether the high-risk residues came from contaminated wildflowers in field margins, other non-focal crops, or other sources is unknown. Yet for regulatory agencies and growers who are interested in minimizing pesticide exposure to bees, understanding the importance of these sources is essential for creating sound management decisions. Current range contractions and loss rates experienced by wild and managed bees are troubling, and the role that pesticides may be playing is controversial. Yet when pesticide risk is found to be high, understanding how to minimize risk is in the interest of both conservationists and growers who rely on bees to provide pollination services.

## Additional Information

**How to cite this article:** McArt, S. H. *et al*. High pesticide risk to honey bees despite low focal crop pollen collection during pollination of a mass blooming crop. *Sci. Rep.* **7**, 46554; doi: 10.1038/srep46554 (2017).

**Publisher's note:** Springer Nature remains neutral with regard to jurisdictional claims in published maps and institutional affiliations.

## Supplementary Material

Supplementary Tables and Figures

## Figures and Tables

**Table 1:** Pesticide residues in recently accumulated beebread collected from experimental honey bee hives at 30 apple orchard sites during bloom (*n* = 30 samples total).

Compound	Compound Type	Mean residue (ppb)^1^	Positive detections	Limit of detection (ppb)^2^	Contact LD_50_ (ug/bee)^3^	Oral LD_50_ (ug/bee)^3^	Mean Contact PHQ^4^	Mean Oral PHQ^4^
Indoxacarb	Insecticide	557.1	2	35.5	0.118	0.26	4721.2	2142.7
Cyfluthrin	Insecticide	93.3	6	35.5	0.037	0.051	2522.8	1830.3
Thiamethoxam	Insecticide	21.5	5	3.6	0.024	0.005	895.3	4297.2
Abamectin	Insecticide	21.9	1	3.6	0.03	NA	729.7	NA
Carbaryl	Insecticide	69.9	11	3.6	0.84	0.15	83.2	466.0
Acetamiprid	Insecticide	160.5	11	1.4	7.9	14	20.3	11.5
Cyprodinil	Fungicide	1216.4	24	0.4	100	100	12.2	12.2
Iprodione	Fungicide	929.3	4	355.3	400	25	9.3	148.7
Thiophanate-methyl	Fungicide	570	1	1.4	100	100	5.7	5.7
Fluxapyroxad	Fungicide	353.6	12	3.6	100	110.9	3.5	3.2
Difenoconazole	Fungicide	327.1	22	1.4	101	177	3.2	1.9
Penthiopyrad	Fungicide	119.2	8	1.4	312	385	3.1	2.5
Atrazine	Herbicide	28.2	6	35.5	97	NA	1.8	NA
Fenbuconazole	Fungicide	389.4	1	35.5	290	NA	1.3	NA
Trifloxystrobin	Fungicide	14.1	18	0.4	200	200	1.3	1.3
Myclobutanil	Fungicide	49.5	1	35.5	39.6	34	1.2	1.5
Fenpyroximate	Insecticide	3.5	1	1.4	11	118.5	0.3	0.0
Thiacloprid	Insecticide	9.6	3	3.6	37.83	17.32	0.3	0.6
Boscalid	Fungicide	0	0	35.5	200	166	0.0	0.0
Phosmet	Insecticide	0	0	355.3	0.62	0.37	0.0	0.0
Clothianidin	Insecticide	0	0	35.5	0.044	0.004	0.0	0.0
Imidacloprid	Insecticide	0	0	3.6	0.044	0.004	0.0	0.0
Spinetoram	Insecticide	0	0	1.4	0.024	0.14	0.0	0.0
Spinosad	Insecticide	0	0	1.4	0.003	0.057	0.0	0.0
Chlorantraniliprole	Insecticide	0	0	3.6	4	104	0.0	0.0

Results are organized in descending order according to mean contact Pollen Hazard Quotient (PHQ).

^1^Mean of positive detections.

^2^Limits of detection (LOD) were determined via a standard dilution series for each individual compound in pesticide-free pollen extraction matrix and assessing the lowest concentration where analyte was detectable.

^3^Contact and oral honey bee LD_50_ toxicity data were obtained from the Tomlin Pesticide Manual^44^, the ECOTOX database of the U.S. Environment Protection Agency (http://cfpub.epa.gov/ecotox/) and the AgriTox Database of the French government (http://www.agritox.anses.fr/index.php). Oral LD_50_ toxicity data was not available for abamectin, atrazine and fenbuconazole.

^4^Pollen Hazard Quotients (PHQ) were determined by dividing total residues (ng/g pollen (ppb)) for each compound by the respective honey bee LD_50_ value (ug/bee)^34^. Mean PHQ values were calculated by summing values for each compound across all sites and dividing by the number of positive detections, thus representing a mean of positive detections.
