# Supplementary material for: High pesticide risk to honey bees despite low focal crop pollen collection during pollination of a mass blooming crop
Source: Sci Rep. 2017 Apr 19;7:46554. doi: 10.1038/srep46554 (PMC5396195; doi:10.1038/srep46554)
Supplement: Supplementary Tables and Figures [file srep46554-s1.pdf]

Supplementary information: High pesticide risk to honey bees despite low focal crop pollen collection during pollination of a mass blooming crop

Authors: Scott H. McArt[a*] shm33@cornell.edu
Ashley A. Fersch[a] aaf72@cornell.edu
Nelson J. Milano[a] njm54@cornell.edu
Lauren L. Truitt[a] llt43@cornell.edu
Katalin Böröczky[b] kb473@cornell.edu

[a] Department of Entomology, Cornell University, Ithaca, NY 14853, USA
[b] Department of Ecology and Evolutionary Biology, Cornell University, Ithaca, NY 14853, USA

[*]**Corresponding author:**
Scott McArt
Email: shm33@cornell.edu
Telephone: 607-255-1377
Fax: 607-255-0939

Table S1. Locations of honey bee colonies that were placed in 30 apple orchards during bloom period (May 7-22, 2015) in western and central New York.

Orchard ID	Longitude	Latitude
A	-76.499255	42.605823
B	-76.65669	42.551575
C	-76.594983	42.573562
D	-76.459711	42.444163
E	-76.568317	42.190409
F	-77.048993	43.247517
G	-77.338395	43.167552
H	-76.472265	42.657503
I	-76.510821	42.358211
J	-77.023644	42.85354
K	-76.550461	42.47103
L	-76.654045	42.562614
M	-77.12573	43.223607
N	-77.106385	43.205716
O	-77.123662	43.169204
P	-77.092533	43.277507
Q	-76.986722	42.832091
R	-76.565643	42.351326
S	-77.22096	43.216111
T	-77.214333	43.217624
U	-77.152612	43.269367
V	-76.523568	42.544315
W	-76.926414	43.198014
X	-77.006645	42.872915
Y	-76.925518	42.377164
Z	-77.006277	42.776101
A1	-76.979654	43.142233
B1	-76.993568	43.203916
C1	-76.860642	43.275884
D1	-76.535329	42.444562

Table S2. Percent apple pollen and total pesticide residues in recently accumulated beebread as predicted by three different landscape variables (percent apple area, natural area and agricultural area) at three spatial buffers (3000, 2000 and 1000 m).

**Landscape variable**	**Response**	**Spatial scale (m)**	**AIC value**	**R^2 value**
Percent apple area	Percent apple pollen	3000	194.75	0.6725*
Percent apple area	Percent apple pollen	2000	196.35	0.6546*
Percent apple area	Percent apple pollen	1000	206.89	0.5091*
Percent apple area	Total pesticides	3000	554.68	0.3664*
Percent apple area	Total pesticides	2000	555.13	0.3564*
Percent apple area	Total pesticides	1000	562.45	0.1719*
Percent natural area	Percent apple pollen	3000	228.58	0.0234
Percent natural area	Percent apple pollen	2000	228.78	0.0168
Percent natural area	Percent apple pollen	1000	229.29	<0.0001
Percent natural area	Total pesticides	3000	568.38	0.0201
Percent natural area	Total pesticides	2000	568.14	0.0281
Percent natural area	Total pesticides	1000	567.78	0.0402
Percent agricultural area	Percent apple pollen	3000	229.28	<0.0001
Percent agricultural area	Percent apple pollen	2000	229.29	<0.0001
Percent agricultural area	Percent apple pollen	1000	229.25	0.0014
Percent agricultural area	Total pesticides	3000	568.59	0.0131
Percent agricultural area	Total pesticides	2000	568.23	0.0253
Percent agricultural area	Total pesticides	1000	567.83	0.0386

* Significant univariate linear model (lm function in R, $\alpha = 0.05$).

Table S3. Pesticides sprayed during apple bloom period (May 7-22, 2015) at the 30 orchard sites, with corresponding toxicity information.

Compound	Class	Type	Contact LD_{50} (ug/bee)[2]	Oral LD_{50} (ug/bee)[2]	Total number of applications*
Abamectin	Macrocyclic lactone	Insecticide	0.03	NA	6
Acetamiprid	Neonicotinoid	Insecticide	7.9	14.0	5
Captan[1]	Dicarboximide	Fungicide	215	91	38
Carbaryl	Carbamate	Insecticide	0.84	0.15	8
Chlorothalonil[1]	Aromatic	Fungicide	135	63	4
Cyprodinil	Pyrimidine	Fungicide	100	100	15
Difenoconazole	Triazole	Fungicide	101	177	18
Dodine[1]	Aliphatic nitrogen	Fungicide	145	NA	2
Emamectin benzoate[1]	Macrocyclic lactone	Insecticide	0.004	NA	6
Fenbuconazole	Triazole	Fungicide	290	NA	4
Fluopyram[1]	Amide	Fungicide	83.2	102.3	5
Indoxacarb	Oxadiazine	Insecticide	0.118	0.26	8
Iprodione	Dicarboximide	Fungicide	400	25.0	3
Glyphosate[1]	Organophosphorus	Herbicide	100	100	2
Lambda-cyhalothrin[1]	Pyrethroid	Insecticide	0.048	0.84	8
Mancozeb[1]	Dithiocarbamate	Fungicide	226	171	46
Oxytetracycline calcium[1]	Antibiotic	Fungicide	>100	NA	2
Pendimethalin[1]	Dinitroaniline	Herbicide	>100	NA	2
Penthiopyrad	Pyrazole	Fungicide	312	385	3
Phosmet	Organophosphate	Insecticide	0.62	0.37	2
Potassium bicarbonate[1]	Inorganic	Fungicide	NA	NA	5
Pyraclostrobin[1]	Pyrazole	Fungicide	100	73	7
Pyrethrins[1]	Pyrethroid	Insecticide	0.14	0.10	1
Pyrimethanil[1]	Pyrimidine	Fungicide	100	100	5
Spinetoram J & L	Macrocyclic lactone	Insecticide	0.024	0.14	1
Spinosad A & D	Macrocyclic lactone	Insecticide	0.003	0.057	2
Thiacloprid	Neonicotinoid	Insecticide	37.83	17.32	1
Thiamethoxam	Neonicotinoid	Insecticide	0.024	0.005	2
Thiophanate-methyl	Benzimidazole precursor	Fungicide	100	100	1
Trifloxystrobin	Antibiotic	Fungicide	200	200	9

[1] Compounds sprayed during bloom but not quantified in pollen (see Table 1).
[2] Contact and oral honey bee LD_{50} toxicity data were obtained from the Tomlin Pesticide Manual [1], the ECOTOX database of the U.S. Environment Protection Agency (http://cfpub.epa.gov/ecotox/) and the AgriTox Database of the French government (http://www.agritox.anses.fr/index.php). Oral LD_{50} toxicity data was not available for abamectin, atrazine, dodine, emamectin benzoate, fenbuconazole, oxytetracycline calcium, pendimethalin and potassium bicarbonate.
* Summed across all 30 sites during bloom period (May 7-22, 2015).

Table S4. Summary of pesticides sprayed during apple bloom (May 7-22, 2015) at each orchard site compared to the number of novel compounds* and risk (measured via contact and oral PHQ [2]) in freshly collected beebread from hives placed at each site during bloom.

Orchard ID	Compounds sprayed at site during bloom	Compounds detected in beebread	Novel compounds* in beebread	Percentage of total contact PHQ attributed to novel compounds*	Percentage of total oral PHQ attributed to novel compounds*
A	6	4	3	99.4	99.9
B	13	3	0	0.0	0.0
C	11	2	1	79.5	87.2
D	8	3	2	29.8	4.3
E	1	1	1	100.0	100.0
F	9	8	5	1.6	33.5
G	5	3	3	100.0	100.0
H	10	6	3	99.9	99.9
I	0	1	1	100.0	100.0
J	12	2	1	13.8	9.2
K	10	3	1	99.0	98.8
L	3	4	4	100.0	100.0
M	10	9	6	92.4	98.1
N	10	7	4	99.4	97.6
O	3	0	0	0.0	0.0
P	6	7	4	92.6	98.6
Q	11	4	2	99.7	99.7
R	5	2	2	100.0	100.0
S	11	3	2	28.4	27.4
T	11	5	2	58.8	84.8
U	8	7	4	0.9	1.0
V	0	3	3	100.0	100.0
W	5	6	4	31.6	72.4
X	0	5	5	100.0	100.0
Y	13	5	4	1.8	0.2
Z	6	3	2	18.3	12.3
A1	9	7	2	0.1	0.2
B1	10	7	5	47.0	75.5
C1	14	11	7	14.4	21.4
D1	2	1	1	100.0	100.0
**Average**	**7.4**	**4.4**	**2.8**	**62.3**	**66.3**

* Pesticides that were not sprayed during bloom at the site but were detected in pollen.

Table S5. Percent of each pollen type identified from recently collected beebread collected from honey bee colonies that were placed in 30 apple orchards during bloom (May 7-22, 2015) in western and central New York.

Site	*Rhamnus* spp.	*Malus/ Pyrus* Type	*Cretaegus /Prunus (prunus)* Type	*Prunus (cerasus)* spp.	*Rubus* Type	*Fragaria* spp.	*Aesculus hippocastanum*	Cichorieae Tribe	*Lonicera* spp.	*Juglans* spp.	Unknown 1	Unknown 2	Unknown 3	Unknown 4	Unknown 5	Unknown 6	Unknown 7	Unknown 8	Unknown 9	Unknown 10	Other
**F**	0.0	34.7	8.7	1.0	11.0	0.0	0.0	0.0	0.3	0.0	1.0	0.7	2.7	1.0	0.3	1.3	0.3	12.0	4.0	0.0	21.0
**P**	0.0	28.0	20.0	4.7	0.7	0.0	0.0	1.0	2.7	0.0	2.0	4.3	1.0	4.0	1.0	0.3	0.0	3.3	0.3	0.0	26.7
**W**	0.0	29.0	6.7	0.0	2.7	0.0	0.0	7.0	5.7	0.3	1.3	1.7	1.7	0.0	3.0	5.0	0.0	8.3	0.3	0.0	27.3
**E**	0.3	4.5	22.5	0.0	0.0	0.0	0.0	1.3	0.0	0.0	0.3	4.5	39.5	0.0	6.4	0.0	0.0	2.9	2.6	0.0	15.1
**H**	0.3	11.0	1.7	0.0	3.0	0.0	0.0	1.3	0.0	0.0	3.0	24.7	14.3	3.3	0.0	3.3	0.0	14.3	0.0	0.0	19.7
**C1**	0.7	33.3	10.0	1.0	0.3	1.3	0.0	1.7	0.3	0.0	2.0	21.7	1.0	1.3	0.0	0.7	0.0	3.0	1.3	0.0	20.3
**B1**	2.7	7.3	2.0	0.0	8.7	0.3	1.0	0.3	1.0	0.0	1.0	17.3	22.7	12.7	0.3	2.7	0.0	1.7	1.7	0.0	16.7
**U**	3.3	19.7	2.0	0.0	0.7	0.3	0.7	0.3	0.3	0.0	8.3	25.0	0.0	13.7	0.0	1.3	0.0	4.0	0.3	0.0	20.0
**R**	4.3	10.3	11.0	0.0	1.3	0.0	0.0	1.7	0.0	0.7	1.7	1.7	7.0	0.0	20.3	4.7	0.0	0.3	0.7	0.0	34.3
**A**	5.0	6.3	1.3	0.3	5.7	0.0	0.0	0.7	0.0	0.0	6.7	36.0	8.7	0.3	3.0	4.7	0.0	0.3	0.0	0.0	21.0
**N**	25.0	4.7	2.3	1.3	1.7	1.3	0.0	0.3	0.0	0.0	1.7	13.0	2.3	10.7	0.3	6.7	0.0	1.0	1.7	0.0	26.0
**Y**	26.7	2.7	8.0	0.0	6.3	0.3	0.7	0.0	0.0	0.0	0.3	28.3	5.3	0.0	9.7	0.7	0.0	0.0	0.3	0.0	10.7
**G**	39.7	0.3	3.0	1.3	4.0	2.7	0.7	0.0	0.3	1.0	0.0	2.7	3.3	3.0	1.0	6.7	0.0	3.3	0.0	0.0	27.0
**I**	41.7	5.3	6.3	0.0	0.7	0.0	11.3	2.0	0.0	0.0	0.0	0.0	3.3	2.0	5.0	3.7	0.0	2.7	2.3	0.0	13.7
**K**	45.3	5.7	3.0	0.0	17.3	0.3	2.0	0.0	0.3	3.3	0.0	0.0	0.0	0.3	6.7	0.3	0.0	0.7	1.3	0.0	13.3
**M**	48.0	11.7	2.0	0.0	0.7	0.0	8.0	0.0	0.3	0.0	1.7	5.3	3.3	0.7	0.0	1.3	0.0	4.3	2.3	0.0	10.3
**T**	48.7	4.0	1.7	0.0	3.0	0.0	1.3	0.0	0.0	0.0	0.7	5.7	8.3	11.3	0.3	2.0	0.0	1.3	1.0	0.0	10.7
**A1**	51.7	11.0	3.3	0.3	0.3	2.7	0.0	0.3	2.0	0.3	2.0	1.3	1.3	2.3	0.3	0.7	0.0	2.3	1.7	0.0	16.0
**S**	55.3	3.7	2.3	0.0	8.0	0.0	0.0	0.3	0.0	0.0	0.7	14.7	2.0	0.7	0.0	1.7	0.0	2.0	0.3	0.0	8.3
**X**	55.7	0.7	0.0	0.0	4.3	0.0	10.0	0.0	0.0	1.3	1.3	0.3	3.3	0.0	0.0	2.0	3.3	1.0	0.3	0.0	16.3
**V**	60.0	14.3	1.3	1.0	0.7	0.0	0.3	0.7	0.7	0.0	0.3	0.0	0.7	0.0	0.7	3.0	0.0	1.0	0.0	0.0	15.3
**Z**	62.0	1.0	0.3	0.0	0.0	0.0	2.0	0.3	0.7	0.0	0.7	20.7	7.3	0.3	0.0	0.0	0.0	0.0	0.0	0.0	4.7
**J**	62.3	0.0	0.0	0.0	0.0	0.0	4.0	0.0	0.3	0.7	4.0	10.7	1.7	5.3	0.0	3.7	0.0	0.0	0.0	0.0	7.3
**O**	66.3	1.7	3.3	0.0	1.0	0.7	0.0	0.0	0.7	0.0	0.3	1.3	4.0	0.0	0.0	0.3	0.0	1.7	0.0	6.3	12.3
**B**	68.3	1.3	0.0	0.0	0.3	1.0	17.7	0.7	0.3	1.7	0.7	0.0	0.0	0.0	0.0	1.0	0.0	0.0	0.0	0.0	7.0
**L**	73.0	1.3	1.0	0.0	2.0	1.3	1.0	0.0	0.3	0.3	0.3	0.0	4.3	0.3	0.3	0.7	0.0	0.0	2.0	0.0	11.7
**Q**	75.3	1.0	0.3	0.0	1.7	0.0	4.7	0.0	0.0	1.0	0.0	1.3	4.7	0.3	0.0	0.0	0.0	0.3	0.3	0.0	9.0
**C**	77.3	5.7	1.7	0.0	1.0	0.0	0.0	0.3	0.3	0.0	0.3	0.0	3.0	0.0	0.7	0.3	0.0	0.0	0.0	0.0	9.3
**D**	78.7	0.7	2.0	0.3	0.0	0.0	0.3	0.0	0.0	0.3	0.7	0.3	1.3	0.3	0.0	4.0	0.0	0.0	0.3	0.0	10.7
**D1**	79.3	1.3	1.7	0.0	2.0	0.3	1.0	0.0	0.0	0.3	0.3	0.3	1.0	0.0	0.7	1.3	0.0	0.0	1.0	0.0	9.3
**Mean**	**38.6**	**8.7**	**4.3**	**0.4**	**3.0**	**0.4**	**2.2**	**0.7**	**0.6**	**0.4**	**1.4**	**8.1**	**5.3**	**2.5**	**2.0**	**2.1**	**0.1**	**2.4**	**0.9**	**0.2**	**15.7**

Fig. S1. Pesticide residues and sources of risk to bees across 30 apple orchard sites. Pollen Hazard Quotient (PHQ) [2] was positively related to total insecticides in beebread (a, red) while there was no relationship with total fungicides (a, blue). There was no relationship Pesticide Use Index (PUI) and insecticides (b, red), fungicides (b, blue), or PHQ (c) when all compounds sprayed during bloom were used to calculate PUI. When only the 25 compounds quantified in beebread were used to calculate PUI, there was a positive relationship between PUI and insecticide residues (d, red), no relationship between PUI and fungicide residues (d, blue), and a positive relationship between PUI and PHQ (e).

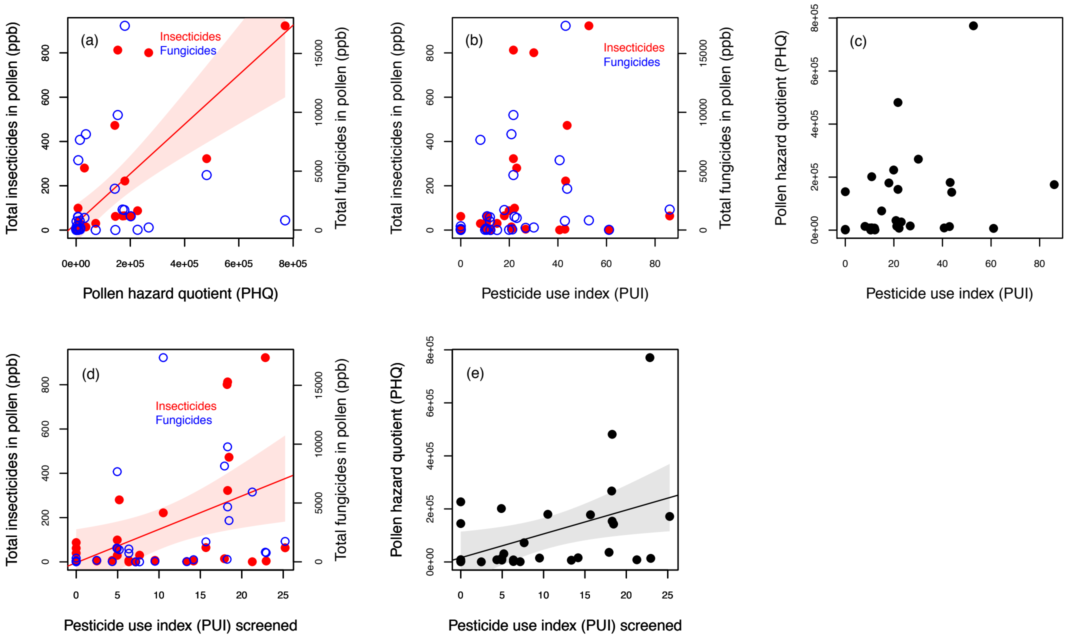

References

1 Tomlin, C. *The Pesticide Manual: A World Compendium, 16th Edition*. (British Crop Protection Council, 2013).

2 Stoner, K. A. & Eitzer, B. D. Using a hazard quotient to evaluate pesticide residues detected in pollen trapped from honey bees (*Apis mellifera*) in Connecticut. *PLoS ONE* **8**, e77550 (2013).
